# Role of Gut Microbiota in Breast Cancer and Drug Resistance

**DOI:** 10.3390/pathogens12030468

**Published:** 2023-03-16

**Authors:** Sathiyapriya Viswanathan, Sheetal Parida, Bhuvana Teja Lingipilli, Ramalingam Krishnan, Devendra Rao Podipireddy, Nethaji Muniraj

**Affiliations:** 1 Department of Biochemistry, ACS Medical College and Hospital, Chennai 600007, Tamil Nadu, India; 2 Department of Oncology, Johns Hopkins University School of Medicine, Baltimore, MD 21231, USA; 3 Gandhi Institute of Technology and Management (GITAM), Deemed University, Visakhapatnam 530045, Andhra Pradesh, India; 4 Department of Biochemistry, Narayana Medical College, Nellore 524003, Andhra Pradesh, India; 5 Rangaraya Medical College, Dr. YSR University of Health Sciences, Kakinada 533001, Andhra Pradesh, India; 6 Center for Cancer and Immunology Research, Children’s National Hospital, 111, Michigan Ave NW, Washington, DC 20010, USA

**Keywords:** microbiome, breast cancer, cancer therapeutics, drug resistance, biomarker and immunotherapy

## Abstract

Breast cancer is the most common malignancy in women worldwide. The cause of cancer is multifactorial. An early diagnosis and the appropriate treatment of cancer can improve the chances of survival. Recent studies have shown that breast cancer is influenced by the microbiota. Different microbial signatures have been identified in the breast microbiota, which have different patterns depending on the stage and biological subgroups. The human digestive system contains approximately 100 trillion bacteria. The gut microbiota is an emerging field of research that is associated with specific biological processes in many diseases, including cardiovascular disease, obesity, diabetes, brain disease, rheumatoid arthritis, and cancer. In this review article, we discuss the impact of the microbiota on breast cancer, with a primary focus on the gut microbiota’s regulation of the breast cancer microenvironment. Ultimately, updates on how immunotherapy can affect the breast cancer-based microbiome and further clinical trials on the breast and microbiome axis may be an important piece of the puzzle in better predicting breast cancer risk and prognosis.

## 1. Introduction

Cancer is the second most common cause of death in the world after heart disease. In 2022, approximately 287,850 cases of breast cancer were diagnosed in women. In addition, approximately 51,400 new cases of breast ductal carcinoma in situ were diagnosed in women [1]. Breast cancer is a heterogeneous disease that is considered to be complex, with great diversity within and between tumors [2]. Despite extensive research, the exact etiology of the disease has not yet been elucidated, as the identified genetic and epigenetic interactions cannot explain the cause of breast cancer in most cases [3]. Therefore, there must be an unexplored pathway that contributes to the development of breast cancer. Studies on cancer risk factors have shown that microorganisms can contribute to the development of cancer in 15–20% of cases [4]. Many of the associations between the gut microbiota and disease are related to both the composition of the microbiota and the specific types of microbes involved in disease development. The relationship between gut microbiota and cancer is unknown.

The human body is a complex symbiotic system of microbial cells and human host cells. There are 100 trillion microbes in our body, distributed everywhere, including the gastrointestinal tract, holding enormous colonies of microorganisms, with their genomes being 150 times larger than the genome of the host cell. These microorganisms comprise the second genome of the human body, and play an important role in health and disease [5,6,7,8]. With advances in sequencing techniques, dysbiotic microbial signatures have been identified that are involved in modulating the onset and progression of various diseases, including cancer [9]. Studies have strong evidence that microbiome composition and regulation, as well as the presence of specific microbes, can initiate tumor formation and promote tumor growth in vivo [10,11,12,13].

Most microbiomes induce cancer through three main mechanisms: 1. altering the balance between cell proliferation and death; 2. controlling the host’s immune system; 3. controlling the host’s metabolism. The microbiota shifts the balance toward cell proliferation by affecting host Wnt/β-catenin, a pathway that maintains cell polarity and growth, and is thus implicated in tumor progression. Proteins such as FadA (*Fusobacterium nucleatum*), Avra (*Salmonella typhi*) and CagA (*Helicobacter pylori*) activate β-catenin, and cause colorectal, hepatobiliary and stomach cancer, respectively [14]. Some bacterial toxins also mediate double-stranded DNA breaks, which the microbiota have developed as a survival mechanism to kill hostile bacteria in its environment. These toxins also mediate cellular DNA damage that leads to carcinogenesis [15]. Colibactin from *Enterococaceae*, Bacteroides fragilis toxin (BFT) from *B. fragilis*, and cytolethal expansive toxin (CDT) from ε/γ proteobacteria are some examples of toxins involved in DNA double-strand breaks [16]. The microbiota can control the host’s immune system and mediate inflammation, which plays an important role in cancer development. Both innate and adaptive immune responses are stimulated by the microbiota [17].

Gut microbiota can alter the efficacy and side effects of cancer treatments. It is clear that the gut microbiota is a double-edged sword in cancer immunotherapy. The gut microbiome contributes to tumor initiation and progression by inducing tumor-promoting inflammation, or by modulating the local tumor microenvironment through its effects on tissue remodeling and mucosal immunity [18]. In addition, it has been suggested that some gut microbes can protect the tumor microenvironment and regulate the anti-cancer immune response, as well as protect the host from inappropriate inflammation. In this review, we provide a broader perspective on the impact of the gut microbiome on the breast cancer microenvironment. Ultimately, the microbiome has direct and indirect effects on cancer immunotherapy, thereby influencing tumor growth and the therapeutic response. Finally, we describe clinical trials targeting the breast cancer and microbiome axis, which may be important for the better prediction of breast cancer risk.

## 2. Microbiome and Breast Cancer, the Connection

According to a 2018 global study, 13% of global cancer burden can be attributed to microbial infections, including both bacterial and viral infection, and this shows a clear geographical association [19]. While the causative agents of cancer were determined to be *H. pylori*, Human papilloma virus, Hepatitis B virus (HBV) and Hepatitis C virus (HCV) [19], it should be noted that the human microbiome is composed of 10–100 trillion microbial partners, most of which remain unidentified. The highest rate of infection associated with cancer development was identified as being in eastern Asia, followed by sub-Saharan Africa, and then by northern Europe and west Asia. China alone accounted for one-third of the cancers driven by *H. pylori* and human papilloma viruses [19]. Therefore, the role of microbes in cancer requires increased attention. Breast cancer is not a single disease, but a variety of different cancers all affecting the breast. Molecular subtyping based on the presence or absence of cell surface receptors, such as ER, PR and Her2, therefore, is used to determine the correct treatment strategy. Patients lacking all three receptors or markers, or triple-negative breast cancer (TNBC) patients, have many adverse outcomes, due to lack of targeted therapies. Breast cancer is a multifactorial disease, potentially impacted by age, lifestyle, parity, exposure to carcinogens and genetics. However, 70% of breast cancers are detected without any known risk factors, other than those of being a woman and being above 50 years of age. Owing to advanced early detection, modern treatment strategies and the public awareness of breast cancer incidence has increased for the past four decades, by 0.5% annually, while mortality has dramatically declined [20]. However, there is an inconsistency in predicting outcomes, particularly among younger and socially disadvantaged women, suggesting that other factors are at play. With the advent of modern sequencing technologies and multicentric techniques, the microbiota has emerged as a potential determinant of breast cancer severity and mortality. Breast cancer can be affected by the local breast microbiota or gut microbiota, either positively or negatively. The microbiota can increase/decrease the risk of breast cancer by regulating circulating steroid hormone levels, regulating energy intake and utilization, synthesizing metabolites, such as genotoxins, lipopolysaccharides, vitamins, and antibiotics, and modulating the immune system.

## 3. The Microbiota of Breast and Breast Tumor

The upsurge in human microbiome research was the direct consequence of the findings of the human microbiome project (HMP), which was initiated in 2007. By 2016, the microbiota of five different body sites of 300 healthy individuals, including the nasal cavity, oral cavity, skin, gastrointestinal tract, and urinogenital tract, was characterized and made publicly available. This marked the beginning of the second phase of the HMP, the integrated HMP (iHMP), which focuses on three non-infectious health conditions, pregnancy and pre-term birth, onset of inflammatory bowel disease and onset of Type 2 diabetes [21]. The breast was considered sterile until Urbaniak et al. proposed the presence of a distinct microbial population in the breast that persisted beyond lactation [22]. Eventually they and other groups, using deep sequencing techniques, such as 16s rRNA sequencing and shotgun sequencing, proved that there is indeed a microbial community living in breast tissue that is significantly altered in breast cancer [23,24]. Moreover, differences were evident between malignant and benign breast cancers and also between different subtypes of breast cancers [24]. Several bacterial genera have been significantly associated with breast cancer. Tzeng, A. et al. examined the 16S rRNA gene sequence of human breast tissues compared to controls. A distinct microbial profile was associated with each histologic tumor subtype; for example, invasive ductal carcinoma (IDC) was characterized by the presence of *Tepidiphilus*, *Alkanindiges*, and *Stenotrophomonas*, while samples of invasive lobular carcinomas (ILC) contained *Peptostreptococcus*, *Micromonospora*, *Faecalibacterium*, and *Stenotrophomonas* [25]. However, their bioinformatic analysis showed that *Porphyromonas*, *Lacibacter*, *Ezakiella*, and *Fusobacterium* were more abundant at a more advanced stage than in lower-stage tumors [25].

Parhi et al. showed that an oral pathogen, *Fusobacterium nucleatum*, could translocate via the blood stream and accumulate in breast tumors, progressively increasing with stages of breast cancer [26]. *F. nucleatum* has been shown to promote breast tumor growth and metastatic progression, possibly by preventing the accumulation of tumor-infiltrating T cells in the tumor microenvironment and colonizing breast tumors through D-galactose-β(1–3)-N-acetyl -D-galactosamine (Gal -GalNAc) which binds to Fap2, a surface lectin from *F. nucleatum,* involved in the colonization of breast cancer. Furthermore, antibiotic therapy with metronidazole suppresses *F. nucleatum*-induced breast tumor aggravation, indicating that targeting *F. nucleatum* may enhance breast cancer treatment [27]. In another detailed study, Parida et al. reported that toxin-producing strains of *Bacteroides fragilis*, when present in the gut or breast tissue, could increase the aggressiveness of breast cancers, induce self-renewal in breast cancer cells and initiate metastatic dissemination to distant organs [10].

The oral administration of *Lactobacillus acidophilus* results in anti-cancer activity in mice bearing breast tumors, via stimulating the Th1 response and enhancing cellular immunity [28]. Another study showed that *Lactobacillus helveticus R389* increased IL-10 and decreased IL-6 levels in serum and mammary cells, thereby suppressing mammary tumor cells by activating the local immune response [29]. Oral administration of *Lactobacillus casei* significantly increased the production of IL-12 and IFN-γ, thereby improving the immune response in mice with invasive ductal carcinoma [30]. In addition, a population-based case–control study showed that the long-term exposure to probiotics, such as *Lactobacillus casei Shirota* and soy isoflavones, protected against breast cancer in Japanese women [31].

Improved imaging techniques, such as fluorescent in situ hybridization and modified PCR protocols, have allowed for the visualization of the spatial organization of microbial riders within the tumor, offering a sneak peek into their potential functions in shaping the tumor microenvironment. In a multicenter study of 1526 tumors and their adjacent normal tissues, Nejman et al. examined nine tumor types, including those in the breast, lung, ovary, pancreas, melanoma, bone, and brain. They demonstrated that tumor-specific microbes resided within tumors, as well as immune cells, in a cell-wall-deficient intracellular state [32]. In addition, breast tumors were found to be the richest and most biodiverse among the nine tumor types examined. In another seminal study, Cai et al. proposed that the internal tumor environment enhanced the metastatic dissemination of breast cancers. The intra-tumor bacteria induce cytoskeletal remodeling in circulating breast cancer cells, making them more resistant to the fluid sheer stress in the circulation, thereby helping them establish colonies in distant sites [33].

## 4. Gut Microbiome and Breast Cancer

A healthy human gut harbors between 300 and 500 bacterial species, predominantly composed of members belonging to four phyla, Actinobacteria, Bacteroidetes, Proteobacteria and Firmicutes [34]. The most important physiological functions, energy assimilation, immune regulation, and xenobiotic metabolism, take place in the gut, and are largely accomplished by the gut microbes [35]. In the context of breast cancer, the gut microbiota plays a complex yet crucial role. In addition to producing pro-carcinogenic toxins, such as BFT from *B. fragilis* and colibactin from pks+ *E. coli*, which can potentially reach the breast tissue via circulation, gut microbes produce metabolites such as cadaverine [36], indoxusulfate [37], and lithocolic acid [38], which are touted to hinder breast cancer progression.

**Figure 1:**
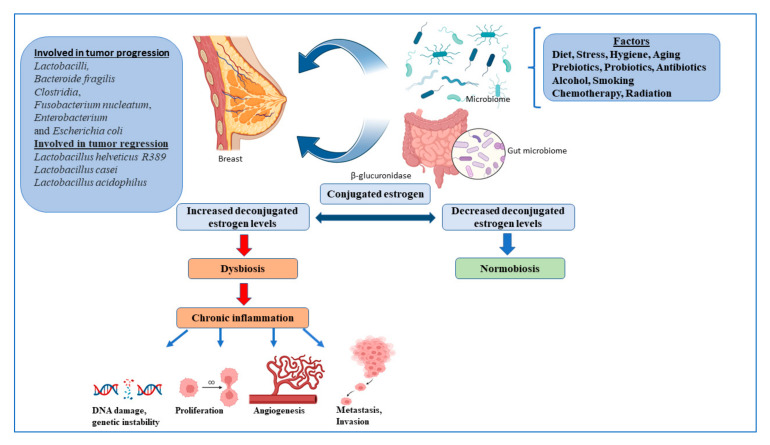
The microbiome and regulation of estrogen in the breast cancer. Figures created with BioRender.com.

Multiple strains of the gut microbes are known to synthesize enzymes that deconjugate conjugated estrogen metabolites, preventing their excretion, and thereby regulating the levels of active estrogens in the circulation, one of the major promoters of breast cancer [39]. Many bacterial species are also known to synthesize estrogen mimics e.g., seasmin, eterolactone and enterodiol, by breaking down dietary lignans [39]. Gut microbial beta glucuronidases convert conjugated estrogen to deconjugated estrogen, which regulates breast dysbiosis, and leads to chronic inflammation, resulting in the alteration of the DNA breaks, proliferation, angiogenesis, metastasis, and invasion (Figure 1).

Gut bacteria act through pathogen-associated molecular patterns (PAMPs), which regulate Toll-like receptors (TLRs) that are responsible for host defense against invading pathogens, and that activate signaling pathways that lead to the induction of immune and inflammatory genes. PAMPs are also responsible for inducing T cells, B cells and CD4 T cells to differentiate into Treg and Th17 cells, which return to the gut or enter the systemic circulation, which can affect immunity at different levels [40]. The gut microbiota supports digestion, metabolism, and host immune responses, resulting in a symbiotic relationship between the host and microbiota, called the normobiosis, that maintains homeostasis [41]. Dysbiosis is caused by changes in the microbiome, leading to a decrease in microbial diversity. As a result, the inability of the microbiota to defend against pathogenic organisms ultimately leads to local and systemic diseases [41]. Obesity, an important breast cancer risk factor, is also closely associated with gut dysbiosis. Multiple studies, to date, have shown significant differences between the gut microbiota of healthy women compared to women with breast cancer, with some showing an overlap with obese microbiota [23,42]. Finally, multiple studies have shown that a healthy gut microbiota is indispensable for the effective utilization of drugs, chemotherapeutics, immunotherapy, and even radiotherapy [40,43].

## 5. Gut Microbiome and Hormone Therapy

Hormone treatment, commonly known as endocrine therapy, is used to treat hormone-sensitive breast cancer. There has been minimal research investigating the relationship between hormone therapy and the gut microbiome in breast cancer. The long-term effects on the microbiome must be determined. Studies reported that some types of breast cancer are hormone-dependent on estrogen and progesterone [44,45,46], but, given the significance of the gut microbiota in estrogen metabolism, this may be of relevance. The gut microbiota varies by race, ethnicity, nutrition, BMI, exposure to antibiotics, and the presence of infections, and it is a major factor in the development of breast cancer [39]. The gut microbiome plays a key role in regulating estrogens, through secretions of β-glucuronidase, an enzyme that deconjugates estrogens into their active forms [39]. The collection of bacteria in the gut responsible for metabolizing and modulating the system’s circulating estrogen comprises an estrobolome. Circulating estrogen levels decline when this process is hindered by gut microbiota dysbiosis, which is defined as a decrease in microbial diversity. Many studies linking dysbiosis of the gut microbiota to many kinds of cancer have been conducted in recent years [47]. Additionally, the circulating estrogens may contribute to pre-menstrual syndrome, obesity, metabolic syndrome, endometriosis, polycystic ovary syndrome (PCOS), infertility, and cardiovascular disease (CVD), due to estrogen dominance.

The consumption of soy products is a major source of isoflavones, which contain phytoestrogens that have been hypothesized to reduce breast cancer risk. Yamamoto S et al. showed that the consumption of miso soup and isoflavones was inversely associated with the risk of breast cancer, in a population-based prospective cohort study in Japan [48]. However, another population-based prospective cohort study suggested that soy and isoflavone intakes have a protective effect on postmenopausal breast cancer in Japan [49]. A probiotic drink containing *Lactobacillus casei* Shirota was found to be inversely associated with breast cancer incidence with the consumption of soy isoflavones [31]. The breast cancer prevention mechanism of soy may be attributed to the estrogenic and antiestrogenic effects of soy isoflavones, such as genistein and daidzein. *Lactobacillus* is a genus of Gram-positive bacteria with the probiotic ability to reduce the incidence of estrogen receptor-positive (ER+) breast cancers, by increasing the anti-cancer activity of tamoxifen and other endocrine system-targeting drugs [50].

## 6. Microbiome and Cancer Immunotherapy

The microbiome has a significant impact on local and systemic host immunity. The microbiota can control the host’s immune system and mediate inflammation, which plays an important role in cancer development. Both innate and adaptive immune responses are stimulated by the microbiota. Cancer immunotherapy has proven to be a promising approach in the treatment of cancer patients. Several studies have demonstrated that the microbiome influences the effectiveness of cancer immunotherapies, especially immune checkpoint inhibitors (ICIs) and CTLA-4 [51]. Microbiome toxicity in response to immune blockades has been studied in animal models. Vetizou M. et al. demonstrated that the anti-tumor efficacy of CTLA blockade depends on the presence of different *Bacteroides* species. T-cell-specific responses to *B. thetaiotaomicron or B. fragilis* correlate with the efficacy of CTLA blockade in mice and patients [13]. Other studies have shown that *Bifidobacterium spp., Ruminococcaceae* and *Faecalibacterium*, found in the gut microbiota, can influence the efficacy of anti-PD-1 immunotherapy in melanoma patients [52,53,54,55]. Supplementation with specific strains of *Bifidobacterium breve* enhances lymphocyte-mediated anti-cancer immunity, thereby inducing efficacy in MC38 colon cancer mice [56].

Patients with metastatic melanoma showed a relative abundance of *Lactobacillales* in the oral microbiome and Bacteroidetes in the fecal microbiome [52]. Patients with a high diversity and abundance of *Ruminococcaceae/Faecalibacterium* have enhanced systemic and anti-tumor immune responses, mediated by increased antigen presentation, as well as improved effector T cell function in the tumor microenvironment [52]. Patients with a higher frequency of Bacteroidetes in the gut microbiome had higher levels of regulatory T cells (Tregs) and myeloid-derived suppressor cells (MDSCs) in the systemic circulation, and impaired cytokine responses. The oral administration of *Bifidobacteria* alone improved tumor control with PD-L1-specific antibody treatment, and combination therapy almost suppressed tumor growth, which may influence the therapeutic response to anti-PD-1 at the level of the tumor microenvironment [54]. Patients with epithelial cancer who were not treated with antibiotics had significantly better overall survival and progression-free survival than those who were treated with antibiotics containing anti-PD-1/PD-L1 [12]. Some challenges to improving the gut microbiome in immunotherapy have been noted, including the presence of unfavorable gut bacteria that may affect the efficacy of immunotherapy, optimal fecal microbial transplantation (FMT) donor selection, and other factors, such as diet, sleep habits, exercise, and medications [51].

An innovative adoptive cell treatment, known as chimeric antigen receptor (CAR) cell therapy, has the potential to alter and instruct immune cells to target certain tumor cells. These CAR-T or CAR-NK cells have a synthetic receptor that is specific to tumor cells expressed on their surface. CARs are divided into four main components, based on their structure and function, including an extracellular ligand-binding domain, most commonly a single-chain variable fragment (scFv), a hinge domain, a transmembrane domain, and an intracellular signaling domain. Over the years, several studies have demonstrated promising therapeutic targets for CAR cell therapy in breast cancer [40,57,58]. Meili Sun et al. demonstrated a novel CAR-T cell therapy for HER2-positive breast and ovarian cancer cells [59]. In another study, Priceman J.S et al. showed that either the intravenous or intraventricular administration of HER2-CAR T cells decreases anti-tumor activity in an orthotropic xenograft model of breast cancer [60]. HER2-specific mouse CAR-T cells increase anti-tumor activity against HER2-positive, transtuzumab-resistant tumor cells in vitro and in vivo [57,58]. A recent study showed that EGFR-CAR-T cell treatment induced a set of immunosuppressive genes through IFN-γ signaling in triple-negative breast cancer cells [61,62]. Zhiwei Hu showed that tissue factor (TF)-CAR-NK enhanced the treatment of TNBC in a xenograft mouse model [63].

## 7. Microbiota Role in Drug Resistance

Chemoresistance is one of the major causes of breast cancer deaths [64]. The microbiota has been shown to play a role in chemoresistance. Chemotherapy and radiation play an integral role in the treatment of almost all types of cancer. Shio et al. reported that the bacterial and fungal microbiota differentially regulate tumor responses to radiation therapy in mouse models of breast cancer. Targeting commensal fungi enhanced the response to radiation therapy and reduced the expression of the C-type lectin receptor Dectin-1, a key innate immune receptor for sensing fungi that contributes to survival in breast cancer [65]. Loss of Dectin-1 abrogated the effect of antifungal agents on radiation therapy. Depleting the bacteria significantly reduces the effect of radiation on tumor growth by curtailing the anti-tumor immune response. Commensal bacteria are required for efficient anti-tumor immune responses, while commensal fungi regulate the immunosuppressive microenvironment following treatment [65]. Several bacterial subsets, including those from the phylum Bacteroidetes and the genera *Bifidobacterium* and *Akkermansia,* have been implicated in regulating the anti-tumor immunity induced by oxaliplatin in colon cancer, and by cyclophosphamide in in vivo fibrosarcoma models [66,67]. Furthermore, *E. coli* was shown to regulate the cytotoxicity of gemcitabine, doxorubicin, etoposide phosphate, etc., in the cancer cell lines [68].

The microbiome particularly affects drug metabolism. Irinotecan (CPT-11), a topoisomerase 1 inhibitor, in combination with fluorouracil and leucovorin, is one of three first-line treatments for metastatic colorectal cancer. FDA approved the use of liposomal irinotecan, as well as 5-fluorouracil and leucovorin, for the treatment of patients with metastatic pancreatic cancer following previous gemcitabine treatment. It is being actively investigated for its use various cancers, including breast cancer [69,70]. Irinotecan is administered to patients intravenously and converted to its active form (SN-38) by carboxylesterases in the liver. SN-38 is inactivated by UDP-glucuronosyltransferases, creating the glucuronidated form (SN-38G), where it enters the intestine via biliary excretion. SN-38G could be reactivated by microbial β-glucuronidases in the gut, which recognize the glucuronidated drug as a carbon source^.^ As a result, adverse drug responses, such as diarrhea, could occur in patients [71]. Breast cancer drugs were shown to regulate microbiota. HER2+ breast cancer patients treated with neoadjuvant trastuzumab (targets HER2) achieved a pathological complete response (pCR), and were characterized by a higher abundance of Clostridiales bacteria and a lower abundance of Bacteroidales. The aromatase inhibitor letrozole, used in treating hormone-positive cancer, resulted in a decrease in the number of Bacteroidales operational taxonomic units (OTUs) and an increase in a majority of Firmicutes OTUs [72,73]. Taken altogether, the potential of microbiota in terms of chemotherapy and radiation needs to be further explored in breast cancer.

## 8. Clinical Trials-Microbiota and Breast Cancer

Currently, several clinical studies are investigating the effect of the microbiota in relation to breast cancer treatment and the quality of life of breast cancer patients (Table 1). However, conclusive clinical trials are needed to demonstrate the benefits of targeting the microbiota in breast cancer.

## 9. Microbiota as a Potential Biomarker in Breast Cancer

The analysis of fecal samples in a pilot breast cancer study revealed that postmenopausal women with newly diagnosed breast cancer had a fecal microbiota that was less diverse and compositionally different when compared with similar women without breast cancer [74]. A comparison of microbiota from 70 women who had breast cancer to healthy individuals revealed that breast cancer patients had a higher relative abundance of *Bacillus*, *Enterobacteriaceae* and *Staphylococcus* when compared to health individuals. Further, *Escherichia coli* and *Staphylococcus epidermidis,* isolated from breast cancer patients, induced DNA double-stranded breaks in HeLa cells [75]. A comparison of 48 postmenopausal breast cancer cases (75% stage 0–I, 88% estrogen-receptor positive) to 48 contemporaneous, postmenopausal, normal-mammogram, age-matched controls revealed that breast cancer cases had significant estrogen-independent associations with the IgA-positive and IgA-negative gut microbiota [76]. An evaluation of 50 ER/PR+, 34 HER2+, 24 ER/PR/HER+, 40 TNBC and 20 heathy breast cancer tissues showed a unique viral, bacterial, fungal, and parasitic signature between the sub-types [77]. Similarly, other studies have shown different microbiota patterns in breast cancer patients [43,78].

## 10. Conclusions

Breast cancer is a multifactorial disease that can be affected by age, lifestyle, parity, exposure to carcinogens and genetic factors. However, 70% of breast cancer cases were found to have no known risk factors, other than being female and over 50 years of age. Accumulating evidence points to a new role for the local immune microbiome in breast cancer. Large-scale studies, including animal models, retrospective and prospective studies, and clinical studies, should be designed to determine the role of the microbiota in breast cancer. This could lead to the identification of anti-tumor microbiomes for the treatment or prevention of breast cancer. All of these investigations, confirming the presence of the microbiota in breast tissue, concentrated on distinct cohorts that included healthy controls vs. breast cancer survivors, benign versus malignant illness, and normal breast versus breast cancer.

## Figures and Tables

**Table 1:** Ongoing clinical trials targeting the microbiome of breast cancer patients.

**Title**	**Clinical Trial No.**	**Study Design**	**Status**
Gut Microbiome Components Predict Response to Neoadjuvant Therapy in HER2-positive Breast Cancer Patients: A Prospective Study	NCT05444647	Observational model: Cohort	Recruiting
Exercise, Gut Microbiome, and Breast Cancer: Increasing Reach to Underserved Populations (EMBRACE)	NCT05000502	Randomized	Recruiting
Assessing the Impact of the Microbiome on Breast Cancer Radiotherapy Toxicity	NCT04245150	Observational model: Cohort	Recruiting
Gut and Intratumoral Microbiome Effect on the Neoadjuvant Chemotherapy-induced Immunosurveillance in Triple Negative Breast Cancer	NCT03586297	Observational model: Cohort	Recruiting
The Association Between Radiation Dermatitis and Skin Microbiome in Breast Cancer Patients	NCT05032768	Observational model: Cohort	Recruiting
Engineering Gut Microbiome to Target Breast Cancer	NCT03358511	Intervention model: Single group assignment	Completed
Evaluating the Association Between Changes in the Gut Microbiome and Chemotherapy-Induced Nausea in Women Receiving Chemotherapy for Stage I-III Breast Cancer	NCT05417867	Observational model: Case-only	Recruiting
Evaluating Mepitel in Post-mastectomy Patients and the Role of the Skin Microbiome in Radiation Dermatitis	NCT03519438	Observational model: Cohort	Completed
Determinants of Acquired Endocrine Resistance in Metastatic Breast Cancer: A Pilot Study (ENDO-RESIST)	NCT04579484	Observational model: Cohort	Recruiting
Oral Aromatase Inhibitors Modify the Gut Microbiome	NCT05030038	Observational model: Cohort	Recruiting
The Breast Cancer Personalized Nutrition Study (BREACPNT)	NCT04079270	Interventional: Randomized	Recruiting
Microbiome and Association With Implant Infections	NCT05020574	Interventional: Randomized (phase 2)	Recruiting
Gut Microbe Composition, Exercise, and Breast Breast Cancer Survivors (ROME)	NCT04088708	Interventional: Randomized	Recruiting
Effect of Radiotherapy Variables on Circulating Effectors of Immune Response and Local Microbiome	NCT03383107	Observational	Completed
Study to Investigate Efficacy of a Novel Probiotic on the Bacteriome and Mycobiome of Breast Cancer	NCT04362826	Interventional: Randomized	Not yet recruited
ARGONAUT: Stool and Blood Sample Bank for Cancer Patients	NCT04638751	Observational model: Cohort	Recruiting
The Gut Microbiome and Immune Checkpoint Inhibitor Therapy in Solid Tumors (PARADIGM)	NCT05037825	Observational model: Cohort	Recruiting
Anti-anxiety Biotics for Breast Cancer Survivors (ABBCS)	NCT04784182	Interventional: Randomized	Completed
Adaptive Nutrition and Exercise Weight Loss (A-NEW) Study (A-NEW)	NCT04499950	Interventional: non-randomized	Recruiting
Effects of Probiotics on the Gut Microbiome and Immune System in Operable Stage I-III Breast or Lung Cancer	NCT04857697	Interventional	Recruiting
Probiotics and Breast Health	NCT03290651	Interventional	Completed
Intestinal Microbiota Impact for Prognosis and Treatment Outcomes in Early Luminal Breast Cancer and Pancreatic Cancer Patients	NCT05580887	Observational model: Cohort	Recruiting
Intestine Bacteria and Breast Cancer Risk	NCT01461070	Observational model: Case-only	Completed
Neoadjuvant Treatment of Locally-advanced Breast Cancer Patients With Ribociclib and Letrozole (NEOLETRIB)	NCT05163106	Interventional	Recruiting
Persistent Post-Surgical Pain in Women With BrCA	NCT02266082	Observational model: Cohort	Completed
Study of Moderate Dose Omega 3 Fatty Acid Supplement in Premenopausal Women at High Risk for Breast Cancer	NCT03383835	Interventional	Un-Known
GRACE-trial: a Randomized Active-controlled Trial for vulvovaginal atrophy in breast Cancer Patients on Endocrine Therapy. (GRACE)	NCT05562518	Interventional: Randomized	Recruiting
Comprehensive Outcomes for After Cancer Health (COACH)	NCT05349227	Interventional: Randomized	Recruiting
Rifaximin for the Treatment of Gastrointestinal Toxicities Related to Pertuzumab-Based Therapy in Patients With Stage I-III HER2 Positive Breast Cancer	NCT04249622	Interventional: Non-Randomized	Recruiting
Impact of Vitamin D Supplementation on the Rate of Pathologic Complete Response in Vitamin D Deficient Patients	NCT04677816	Interventional: Non-Randomized	Recruiting
Weight Loss Plus Omega-3 Fatty Acids or Placebo in High Risk Women	NCT02101970	Interventional: Randomized	Recruiting
Comprehensive Lifestyle Change To Prevent Breast Cancer	NCT03448003	Interventional: Randomized	Recruiting
Avera/Sema4 Oncology and Analytics Protocol (ASAP)	NCT05142033	Interventional: Randomized	Recruiting
Gender Difference in Side effects of Immunotherapy: A Possible Clue to Optimize Cancer Treatment (G-DEFINER)	NCT04435964	Observational	Recruiting
Neoadjuvant Pembrolizumab(Pbr)/Nab-Paclitaxel Followed by Pbr/Epirubicin/Cyclophosphamide in TNBC (NIB)	NCT03289819	Interventional	Completed
Abemaciclib in Treating Patients With Surgically Resectable, Chemotherapy Resistant, Triple Negative Breast Cancer	NCT03979508	Interventional: Non-Randomized	Recruiting

The clinical trials information obtained from https://clinicaltrials.gov/.

## Abbreviations

BFT	*Bacteroides fragilis* toxin
HBV	Hepatitis B virus
BCV	Hepatitis C virus
ER	Estrogen receptor
PR	progesterone receptor
TNBC	Triple negative breast cancer
HMP	Human microbiome project
iHMP	Integrated Human microbiome project
IDC	Invasive ductal carcinoma
ILC	Invasive ductal carcinoma
PAMPs	Pathogen-associated molecular patterns
ICIS	Immune checkpoint inhibitors
T-regs	Regulatory T cells
FMT	Fecal microbial transplantation
pCR	Pathological complete response
OTUs	Operational Taxonomic Units
PCOS	Polycystic ovary syndrome
CVD	Cardiovascular disease
